# Hypertensive crisis in the critically ill

**DOI:** 10.1016/j.aicoj.2026.100158

**Published:** 2026-09-14

**Authors:** Julien Demiselle, Nans Florens, Sabrina Garnier-Kepka, Antonin Trimaille, Hamid Merdji

**Affiliations:** a Service de Médecine Intensive Réanimation, Hôpital de Hautepierre, Hôpitaux Universitaires de Strasbourg, France; b INSERM UMR 1260 Nanomedicine Regenerative, France; c Groupe INTERFERENCE, Nouvel Hôpital Civil, Hôpitaux Universitaires de Strasbourg, France; d Service de Néphrologie, Dialyse, Transplantation Nouvel Hôpital Civil, Hôpitaux Universitaires de Strasbourg, France; e INSERM UMR S_1109, Fédération Hospitalo‑Universitaire TARGET, FMTS, University of Strasbourg, Strasbourg, France; F-CRIN, INI CRCT, Nancy, France; f Service des Urgences, Hôpitaux Universitaires de Strasbourg, France; g ICube Illkirch, UMR 7357 équipe IMAGeS, University of Strasbourg, France; h Service de Cardiologie, Nouvel Hôpital Civil, Hôpitaux Universitaires de Strasbourg; i UR 3074, Translational Cardiovascular Medicine, CRBS, University of Strasbourg, Strasbourg, France; j Service de Médecine Intensive Réanimation, Nouvel Hôpital Civil, Hôpitaux Universitaires de Strasbourg, France

**Keywords:** Hypertensive crisis, Hypertensive emergency, Malignant hypertension, Intensive care unit, Antihypertensive agents

## Abstract

Hypertensive crises encompass a spectrum of clinical presentations ranging from severe asymptomatic blood pressure (BP) elevation to life-threatening hypertensive emergencies and represent a frequent and challenging situation in the critically ill.

While hypertensive urgency, defined as a marked elevation in blood pressure without acute organ damage, can generally be managed in the outpatient setting, hypertensive emergencies require immediate in-hospital management. Hypertensive emergencies are defined by a BP ≥180/110 mmHg associated with acute target-organ damage. Malignant hypertension represents a distinct and severe subset, further characterized by microvascular complications including hypertensive retinopathy and fibrinoid necrosis affecting multiple organs.

Initial evaluation is critical to decipher between hypertensive urgency and emergency, with a systematic screening for end-organ damage: cardiovascular system (heart, aorta, and large vessels), central nervous system, retina, and kidneys. The acuity of BP rise, rather than its absolute level alone, largely determines the risk of organ injury, underscoring the importance of a thorough clinical and paraclinical assessment.

Once a hypertensive emergency is identified, prompt initiation of intravenous antihypertensive therapy is mandatory. Drug selection, target BP, and the rate of BP reduction must be individualized according to the specific type of end-organ damage.

In this narrative review, we summarize current definitions and epidemiology of hypertensive emergencies, detail the clinical approach to initial assessment, and provide an etiology-based overview of management strategies with a focus on intravenous antihypertensive agents and BP targets. Finally, we emphasize the importance of structured long-term follow-up after the acute phase, including etiological workup and optimization of antihypertensive therapy, to prevent recurrence and target-organ failure.

## Graphical abstract

**Figure:**
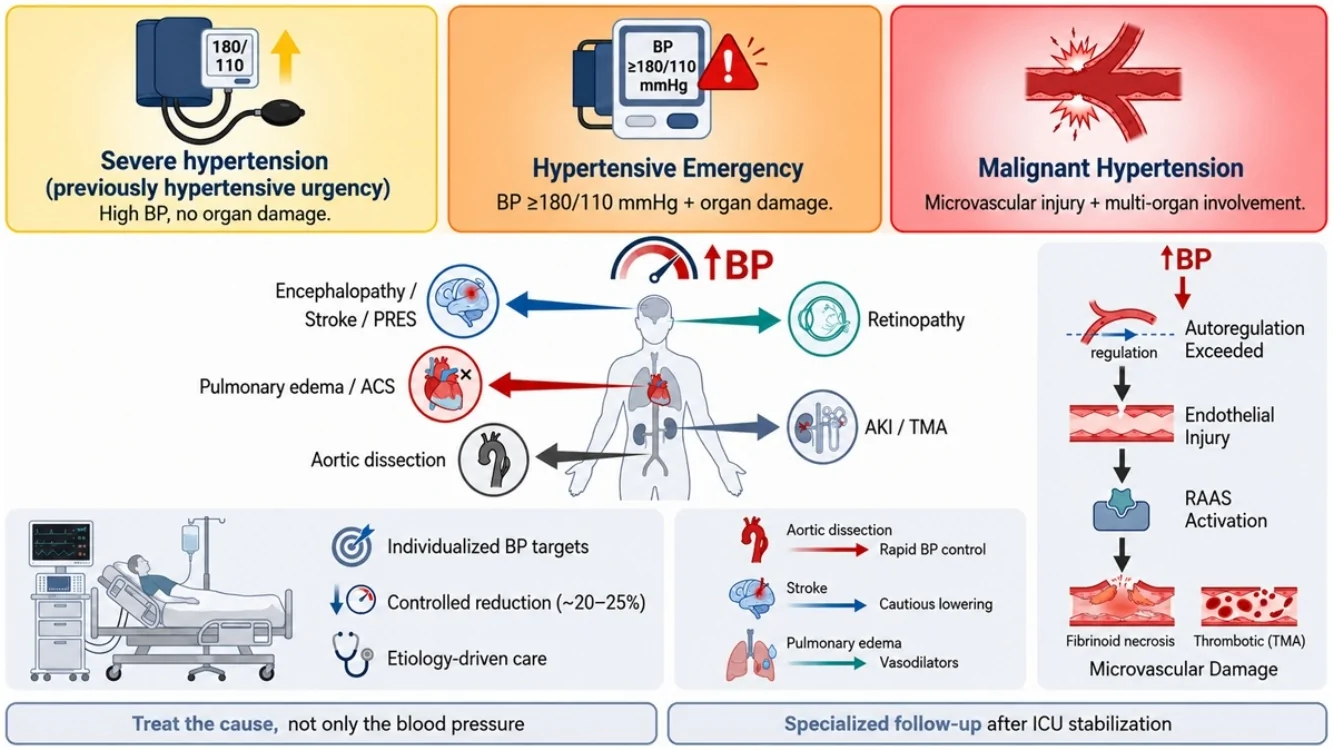

## Background

The term “hypertensive crisis” has been used in the literature to describe severe hypertension, both with and without associated target organ damage. According to the latest consensus from the **European Society of Cardiology (ESC)** [1] and **North American (AHA/ACC)** guidelines [2], the following distinctions should be made:

• **Severe hypertension** (formerly referred to as hypertensive urgency): Defined as a systolic blood pressure (BP) greater than 180 mmHg and/or diastolic BP greater than 110 mmHg [1] or 120 mmHg [2], without clinical evidence of acute organ damage. Although these patients require BP reduction, they typically do not require hospitalization and are therefore beyond the scope of this review. This presentation is also called “asymptomatic markedly elevated BP” in a recent scientific statement from the American Heart Association [3].
• **Hypertensive emergency:** Defined as a BP ≥180/110 mmHg in the European guidelines [1] and ≥180/120 mmHg in the North American guidelines [2], in association with acute target-organ damage and often accompanied by clinical symptoms.
• **Malignant hypertension:** Defined as a hypertensive emergency and evidence of acute microvascular damage (microangiopathy), such as fibrinoid necrosis, affecting the microcirculation of target organs [1]. Among damaged organs, hypertensive retinopathy is a frequent condition.

Initial assessment of patients with severe hypertension aims to stratify risk, identifying those likely to develop, or who have already developed, a hypertensive emergency, defined by the presence of acute target organ injury. Conversely, patients presenting with severe hypertension, without evidence of acute target organ injury, can often be managed in the outpatient setting. After confirming the BP elevation and investigating potential triggers (e.g., medication non-adherence, stress, intoxication, pain, etc.), oral antihypertensive therapy can be initiated, along with close follow-up. Such medical cases fall outside the scope of this review.

This distinction underscores the critical importance of end-organ damage assessment.

It is noteworthy that diagnosing a hypertensive emergency remains clinically challenging. Although the rapidity of blood pressure elevation, rather than its absolute level alone, is an important determinant of acute organ injury, it is rarely known at presentation. Furthermore, structural or functional organ abnormalities identified at admission may reflect pre-existing chronic disease rather than an acute process.

In this narrative review, we detail epidemiology, pathophysiology and the initial patient’s evaluation. Then, we will provide an etiology-based overview of management strategies with a focus on intravenous antihypertensive agents and BP targets.

## Hypertensive emergencies

Hypertensive emergencies are diagnosed when both significantly elevated BP and acute target organ damage are present. Specific BP criteria may differ slightly, and are defined as BP:

• ≥180/110 mmHg according to the latest European guidelines [1].
• >180/120 mm Hg according to the latest United States guidelines [2].
a Epidemiology:

Epidemiological data on hypertensive emergencies encompasses a wide spectrum of etiologies and patient populations, with definitions and typologies that may vary across studies. A previous single-center prospective Italian study over 12 months reported that significant BP elevations accounted for 11% of emergency department admissions, with hypertensive emergencies comprising 27% of these cases [4] Notably, the incidence of hypertensive emergencies has been increasing in countries with readily accessible healthcare [5,6]. A prospective study of over 1500 patients in the United States admitted in intensive care units (ICU) for hypertensive emergencies provided insights into patient demographics and clinical characteristics. The median age was 58 years, with an equal sex distribution. Cardiovascular and renal comorbidities were prevalent, with 31% of patients having a history of chronic kidney disease. The majority of the cohort (56%) was African American. Medication non-adherence was reported in 25% of the patients [7].

a Clinical Presentation and Assessment of End-Organ Damage

Standardized BP measurement, as outlined in international guidelines [1], is essential for accurate assessment. This involves using a cuff size tailored to the patient's arm circumference, ensuring the patient has rested for at least 5 min in a seated position, and avoiding exertion, caffeine, or tobacco use within 30 min before measurement. Three readings should be taken (1−2 min apart), and the average of the last two measurements should be used for analysis. However, this protocol is rarely feasible in acute scenarios. For these high-acuity presentations, clinicians should initially verify BP through rapid serial measurements in both arms to rule out acute aortic dissection, before transitioning quickly to continuous invasive intra-arterial monitoring. As stated in the AHA scientific statement, arterial lines are preferred for hypertensive emergencies and for intravenous antihypertensives [3]. Indeed, studies have found that when BPs exceed 180/100 mmHg in critical care or surgical inpatient populations, oscillometric devices may underestimate systolic and diastolic values by as much as 50/30 mmHg compared with direct arterial line measurements [8,9]. Of note, the findings of the recent EVERDAC trial, which demonstrated the noninferiority of a strategy without early arterial catheter insertion compared with early insertion in critically ill patients, may not be directly generalizable to critically ill patients with hypertensive crises, as the trial population comprised exclusively patients with circulatory shock [10].

Once severe hypertension is confirmed, the clinical assessment should focus on identifying end-organ damage, which commonly affects the cardiovascular system (heart, aorta, and large vessels), retina, central nervous system, and kidneys [5,11] (Fig. 1). Clinical examination should specifically assess for:

• **Cardiac involvement:** orthopnea, dyspnea at rest, crackles, angina, electrocardiographic evidence of ischemia
• **Vascular involvement:** migrating chest pain, asymmetric pulses
• **Neurological involvement:** headache, visual disturbances, confusion, vertigo. In patients with hypertensive encephalopathy, somnolence, lethargy, tonic-clonic seizures, and cortical blindness may precede loss of consciousness; however, focal neurological deficits are rare and should raise suspicion for stroke.
• **Renal involvement:** proteinuria (detected by urine dipstick) and acute kidney injury.

**Fig. 1:**
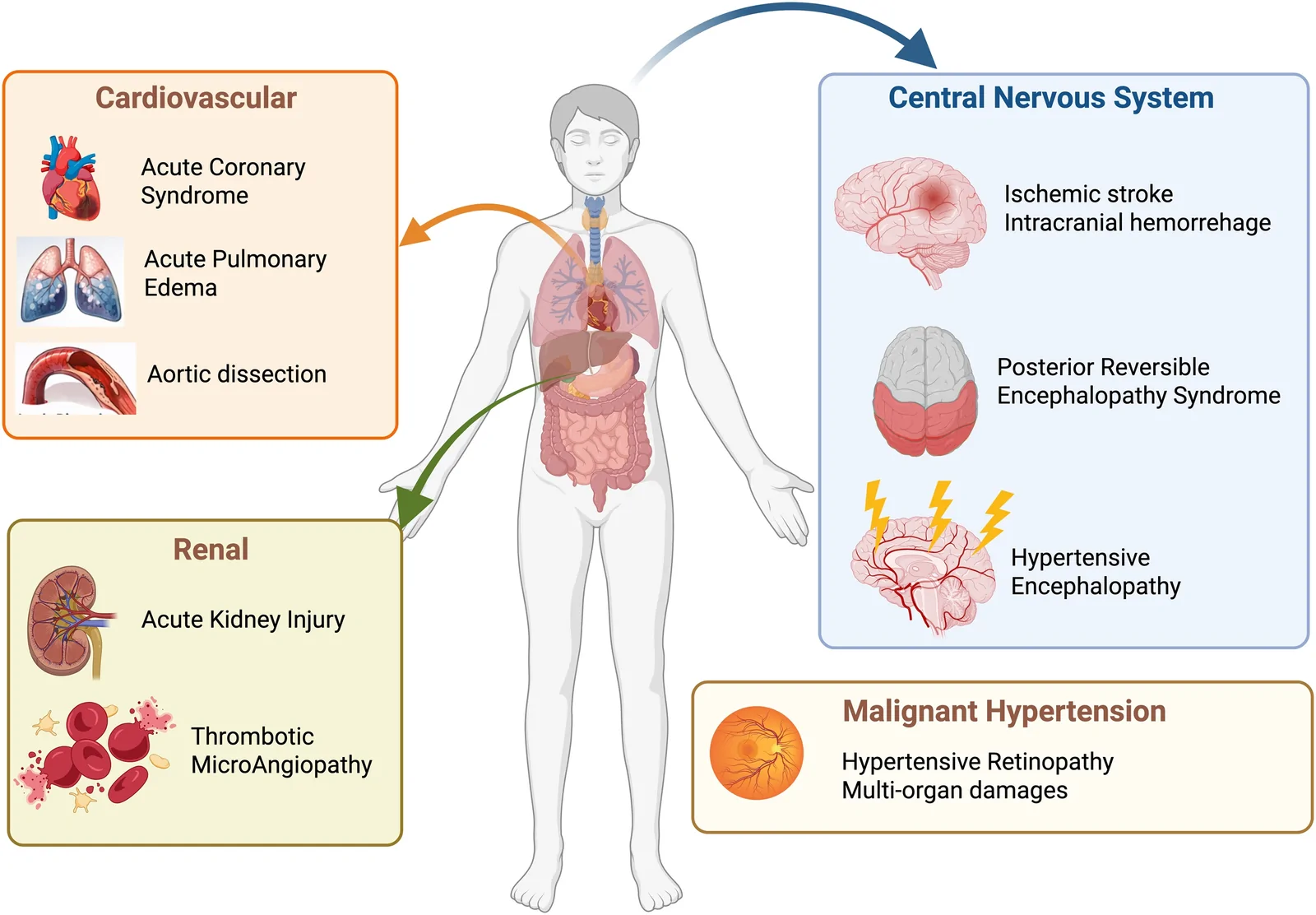
Main organ-targeted involved in the course of hypertensive emergencies (except pregnancy situations).

A minimal paraclinical workup is recommended (Table 1), with fundoscopy strongly encouraged, although accessibility may be limited in the acute setting [1].

**Table 1:** Diagnostic work-up for patients with a suspected hypertensive emergency, according to [1].

Common tests for all potential causes:
▪ Fundus examination ▪ 12-lead ECG ▪ Hemoglobin, platelets count, fibrinogen ▪ Creatinine, eGFR, electrolyte, LDH, haptoglobin ▪ Urinary albumin-to-creatinine ratio, urine microscopy for red cells, leucocytes, casts ▪ Pregnancy test in women of childbearing age
Specific tests by indication:
▪ Troponin, CK-MB (in suspected cardiac involvement, e.g. acute chest pain or acute heart failure), and NT-proBNP ▪ Chest X-ray (fluid overload) ▪ Echocardiography (aortic dissection, heart failure, or ischemia) ▪ CT angiography of thorax and/or abdomen in suspected acute aortic disease (e.g. aortic dissection) ▪ CT or MRI brain (nervous system involvement) ▪ Renal ultrasound (renal impairment or suspected renal artery stenosis) ▪ Urine drug screen (suspected methamphetamine or cocaine use)

In the presence of target organ damage, prompt hospitalization and intravenous antihypertensive therapy are required to rapidly lower BP and mitigate further damage.

c Management

An essential component of prescribing intravenous antihypertensive agents involves accurately determining the target BP levels (systolic/diastolic) (Fig. 2), selecting the most suitable drug class, establishing the optimal dosing regimen, and determining the frequency of reassessment, to ensure precise and efficient administration.

**Fig. 2:**
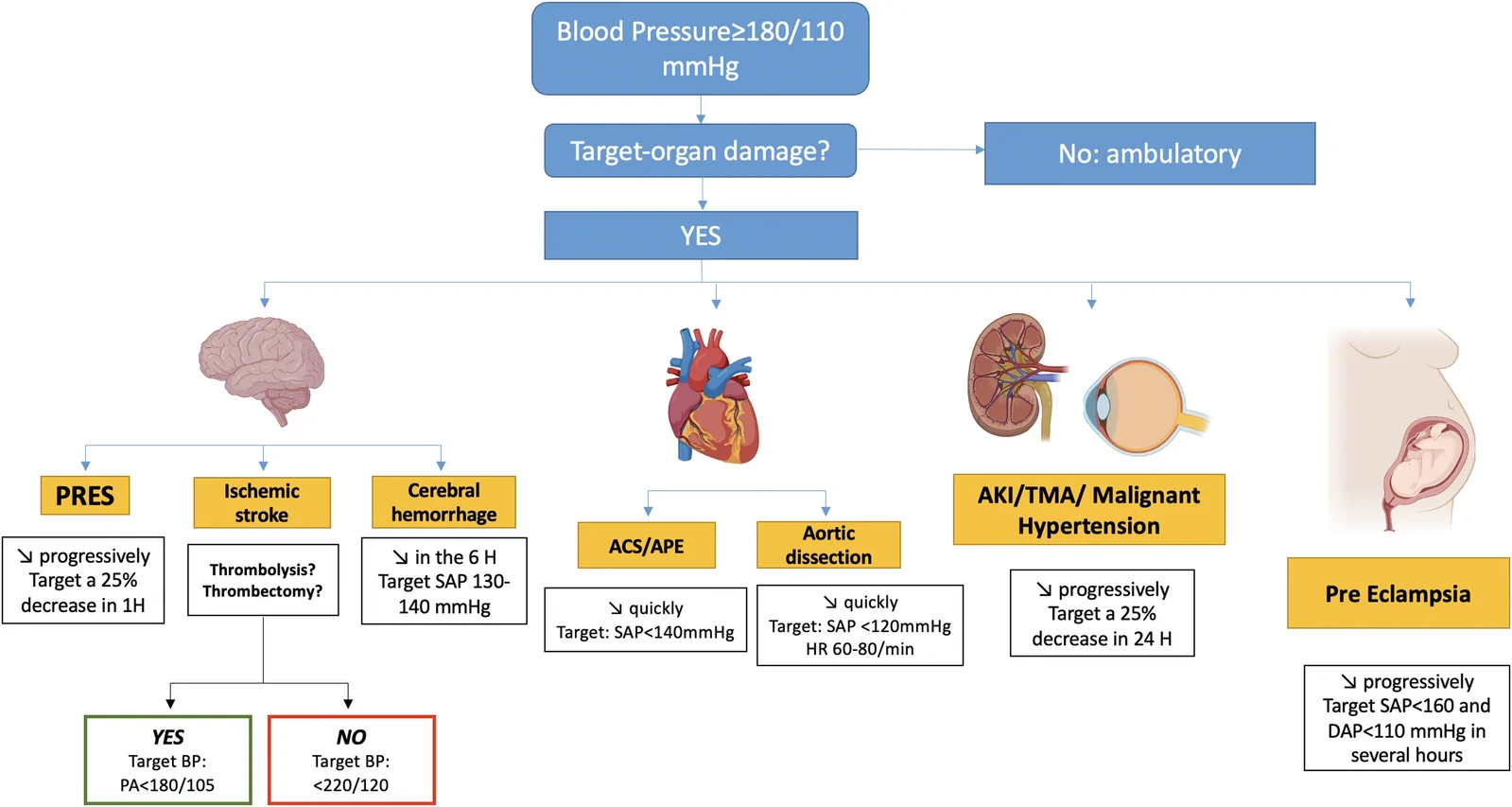
Blood pressure management according to the clinical scenarios of hypertensive emergencies. ACS: Acute Coronary Syndrome, AKI: Acute Kidney Injury, APE: Acute Pulmonary Edema, BP: Blood Pressure, HR: Heart Rate, DAP: Diastolic Arterial Pressure, PRES: Posterior Reversible Encephalopathy Syndrome, SAP: Systolic Arterial Pressure, TMA: Trhombotic Microangiopathy.

Table 2 provides an overview of the key antihypertensive agents, including their common dosages, routes of administration, and relevant pharmacokinetic properties.

i Cardiovascular Injuries

**Table 2:** Pharmacological properties, mechanisms, and clinical indications of main intravenous antihypertensive agents for the treatment of hypertensive emergencies, according to [1].

Drug (Class)	Posology	Pharmacodynamics	Mechanism of action & hemodynamic effects	Primary clinical indications	Precautions & adverse events
Nicardipine (Dihydropyridine Calcium Channel Blocker)	• No initial bolus • Continuous infusion: 5−15 mg/h (titrated max15 mg/h)	• Onset: 2–10 min • Duration of action: 30–40 min	• Predominantly dilates resistance arterioles, thereby reducing systemic vascular resistance and left ventricular afterload, with little direct effect on venous capacitance or cardiac preload. • Heart Rate: May induce mild to moderate reflex tachycardia and generally maintains or increases cardiac output because of afterload reduction. • Coronary/Cerebral: Dilates coronary and cerebral resistance vessels, preserving or improving regional blood flow.	• Acute Ischemic Stroke (pre- or post-thrombolysis/thrombectomy) • Intracerebral Hemorrhage • PRES (with hypertension)/ Hypertensive Encephalopathy • Malignant Hypertension • Acute Aortic Syndromes • Severe Preeclampsia (second-line/add-on)	Reflex tachycardia, headache. Preservation or improvement of cerebral blood flow cannot be assumed if systemic blood pressure is reduced excessively, particularly in patients with impaired cerebral autoregulation. Increases calcineurin inhibitor blood levels (cyclosporine/tacrolimus overdose risk). Contraindicated in advanced hepatic failure.
Urapidil (α–1 blocker and central antihypertensive)	• Bolus: 12,5–25 mg IV • Continuous infusion: 5–40 mg/h	• Onset: 3–5 min • Duration of action: 4–6 h	• Reduces systemic vascular resistance and left ventricular afterload through peripheral α₁-adrenergic receptor blockade. • Central 5-HT₁A receptor agonism reduces sympathetic outflow and generally attenuates reflex tachycardia. • Cerebral perfusion is generally maintained during controlled blood pressure reduction but may decrease if excessive hypotension occurs.	• Acute hypertensive crises requiring stable heart rate control • Intracerebral Hemorrhage (add-on to Calcium Channel Blockers) • Malignant Hypertension	Dizziness, nausea, transient fatigue. IV continuous use should generally not exceed 7 days (longer durations have not been adequately evaluated in toxicological studies).
Nitroglycerin (Direct Nitric Oxide (NO) Donor)	• Infusion: 5–200 μg/min	• Onset of action: 1–5 min • Duration of action: 3–5 min	• Venous > Arterial Vasodilator: Predominantly reduces venous preload at lower doses; decreases afterload at higher doses. • Coronary/Cerebral: Selective dilation of large epicardial coronary arteries; can increase intracranial pressure via cerebral venodilation.	• APE • AMI • Acute Aortic Syndromes (as second-line adjunct to beta-blockers)	Avoid in Right Ventricular Infarction (cardiac output is dependent on adequate preload). Risk of reflex tachycardia (increases MVO_2_), headache, tolerance during prolonged infusion.
Isosorbide dinitrate (Organic nitrate)	• Infusion: 2–10 mg/h	• Onset of action: 1–2 min • Duration of action: 30–60 min after a single IV dose, but potentially several hours after prolonged infusion because of active-metabolite accumulation (Note: active metabolite IS-5-MN has half-life of 4–6 h, leading to long-lasting systemic effects)	• Venous > Arterial: At low dose, reduce venous return, right- and left-sided filling pressures, pulmonary capillary wedge pressure and cardiac preload. • At higher doses, arterial and arteriolar dilation additionally reduces systemic vascular resistance and left ventricular afterload. • Dilates epicardial coronary arteries and relieves vasospasm May cause cerebral vasodilation and potentially increased intracranial pressure	• APE • AMI • Acute Aortic Syndromes (as second-line adjunct to beta-blockers)	Avoid in Right Ventricular Infarction (cardiac output is dependent on adequate preload). Risk of reflex tachycardia (increases MVO_2_), headache, tolerance during prolonged infusion. Prolonged infusion may increase the risk of sustained hypotension because of the accumulation of pharmacologically active metabolites.
Labetalol (Non-selective β-blocker and α–1 blocker)	• Bolus: 0,25−0,5 mg/kg over 1 min • Continuous infusion: 2–4 mg/min	• Onset of action: 5–10 min • Duration of action: 3–6 h	• Reduces systemic vascular resistance and afterload through α₁ blockade. Reduces or limits heart rate and myocardial contractility through β₁ blockade. Usually maintains cardiac output because the reduction in afterload offsets its negative chronotropic and inotropic effects. • Generally maintains cerebral and uteroplacental perfusion when BP reduction is gradual and appropriately titrated.	• Severe Preeclampsia & Eclampsia (first-line) • Acute Ischemic Stroke • Intracerebral Hemorrhage • Malignant Hypertension • Acute Aortic Dissection (when esmolol is unavailable)	Adverse effects: Hypotension, bradycardia, atrioventricular block, bronchospasm and worsening or precipitation of acute heart failure. Contraindications: Severe bradycardia, second- or third-degree AV block in the absence of a functioning pacemaker, cardiogenic shock and bronchial asthma or severe active bronchospasm.
Esmolol (Ultra-short-acting selective β1-blocker)	• Bolus: 0.5–1 mg/kg over 1 min • Continuous infusion: 50–300 µg/kg/min	• Onset of action: 1–2 min • Duration of action: 10–30 min	• Rapidly reduces heart rate, myocardial contractility and the rate of rise of left ventricular pressure (dP/dt), thereby reducing cardiac output and MVO_2_. • Reduces the frequency and force of left ventricular ejection, thereby decreasing aortic-wall stress and shear forces. Minimal direct vasodilatory activity.	• Acute Aortic Syndromes (Aortic Dissection/Intramural Hematoma) (first-line)	Hypotension, bradycardia, AV block, reduced cardiac output and worsening acute heart failure. Contraindicated in severe bradycardia, second- or third-degree AV block without a functioning pacemaker, cardiogenic shock. Use cautiously in severe reactive airway disease. In pheochromocytoma, esmolol must not be administered before adequate α–adrenergic blockade because of the risk of unopposed α–adrenergic vasoconstriction and hypertensive deterioration.

**Acute pulmonary edema (APE):** hypertension is a significant pathophysiological factor contributing to APE due to the increased left ventricular afterload. Hypertension can be both a triggering factor and an aggravating factor for left ventricular failure. The 2026 ESC guidelines recommend intravenous nitrates (i.v. nitroglycerine or nitroprusside), as first line therapy for hypertensive acute heart failure, primarily to reduce left ventricular afterload. They further state that intravenous vasodilators should be considered as initial therapy in patients with systolic BP >110 mmHg to alleviate symptoms, reduce congestion and decrease the need for tracheal intubation (Class IIb, Level C). However, available evidence has not demonstrated that these therapies reduce mortality or length of hospital stay in patients with decompensated heart failure (DHF) [12]. Moreover, intravenous loop diuretics are recommended for all patients with DHF admitted with fluid overload to improve symptoms (Class I, Level A) [12]. Notably, some diuretics, such as furosemide, exhibit slight vasodilatory properties [13,14]. Despite the lack of a clearly defined BP target for APE in current literature, a systolic BP (SBP) below 140 mmHg is generally advocated [15]. A recent scientific statement from the Heart Failure Association (HFA) of the ESC further suggests an initial SBP reduction of 25% within the first hour, aiming for a target SBP of 130−140 mmHg by 6 h.

**Acute coronary syndrome:** The evaluation for acute myocardial infarction (AMI) is crucial, as hypertension exacerbates myocardial oxygen demand (MVO_2_) by increasing afterload, thereby contributing to myocardial ischemia. While there is no specific literature recommending a particular antihypertensive agent or BP target in primary AMI [16], a systolic BP goal of less than 140 mmHg is often suggested in practice [15]. Intravenous nitrates may be beneficial in the acute phase of AMI, however, caution is mandatory as they can induce a harmful reflex tachycardia (increasing MVO_2_). Additionally, they must be avoided in right ventricular infarction, a clinical scenario where cardiac output relies heavily on an intact preload [12].

Secondary myocardial infarction (formerly referred to as type 2 AMI), represents a further therapeutic challenge [16]. Severe hypertension may precipitate it by increasing left ventricular afterload and MVO2, particularly in patients with underlying coronary artery disease or left ventricular hypertrophy. Evidence specifically guiding BP management in this setting remains limited. Treatment should primarily address the precipitating supply–demand imbalance. When severe hypertension is identified as a precipitating or aggravating factor, prompt but carefully titrated antihypertensive therapy should be initiated, while avoiding excessive reductions in diastolic blood pressure that could compromise coronary perfusion [17].

**Acute aortic syndromes:** Severe hypertension accompanies aortic dissections (the most common acute aortic syndrome), intramural hematoma, and penetrating atherosclerotic ulcer. Control of both BP and heart rate is essential to limit the extension of the dissection by reducing the wall stress induced by hypertension and shear forces. In cases of aortic dissection, a rapid reduction in systolic BP to a target strictly below 120 mmHg with a heart rate between 60 and 80 beats per minute is recommended. Short-acting beta-blockers such as esmolol are preferred, with the addition of calcium channel blockers or nitrates if necessary to achieve the target BP [18].

ii Central Nervous System Injuries

**Hypertensive encephalopathy and posterior reversible encephalopathy syndrome (PRES):** The clinical presentation of hypertensive encephalopathy varies and may include generalized headaches, nausea, vomiting, visual disturbances, and significant fatigue, depending on the severity. Agitation and/or impaired higher cognitive functions may subsequently develop. Coma, often accompanied by generalized or focal seizures, may occur. Fundoscopic examination may reveal exudates, hemorrhages, and papilledema [19].

PRES, a specific type of hypertensive encephalopathy, is primarily caused by hypertension [20]. The pathophysiology of both conditions is similar: a significant elevation in BP that exceeds cerebral autoregulation leads to increased cerebral blood flow and perfusion pressure, resulting in plasma extravasation across the blood-brain barrier and vasogenic brain edema [19]. The predilection of PRES to affect the posterior parietal and occipital lobes may be due to the relatively sparser sympathetic innervation in these areas. This limits adaptive vasoconstriction during acute BP spikes, rendering these posterior regions highly susceptible to breakthrough hyperperfusion, endothelial injury, and vasogenic edema [21].

However, the absence of hypertension does not exclude PRES, as 20%–40% of patients may be normotensive at presentation, particularly in the context of endothelial dysfunction, sepsis, autoimmune disease, eclampsia or exposure to cytotoxic or immunosuppressive therapies [22].

Patients with PRES typically present with severe hypertension (usually >220/120 mmHg) and neurological symptoms, often visual disturbances such as blurred vision, photopsia, visual field defects, or cortical blindness, which may progress to seizures (partial or generalized) [19]. Characteristic MRI findings include T2/FLAIR hyperintensities predominantly in the parietal and occipital lobes, although other locations may be involved [23]. While MRI remains the gold standard, its availability in emergent situations may be limited. In such cases, contrast-enhanced cerebral CT scan should be promptly performed to evaluate for alternative diagnoses, including but not limited to ischemic stroke, central nervous system infections, demyelinating diseases, brain tumors, dural venous sinus thrombosis, central nervous system vasculitis, toxic encephalopathy, and reversible cerebral vasoconstriction syndrome.

Intracerebral hemorrhage is a potential complication of PRES, occurring in 10–25 % of cases [24].

Management requires intravenous antihypertensive therapy, although no specific strategy has been definitively established. Intravenous calcium channel blockers are commonly used, although caution is warranted with nicardipine in immunosuppressed patients receiving calcineurin inhibitors because of potential pharmacokinetic interactions. Central-acting antihypertensive agents may also be considered. The initial objective is to reduce BP by approximately 25% within the first hour, while ensuring adequate cerebral perfusion and minimizing the risk of ischemic complications. Subsequently, gradual normalization of BP should be achieved within 24−48 hours [2]. In cases of hemorrhagic complications, management should probably be based on the elements discussed below.

Hypertensive encephalopathy is associated with an increased risk of subsequent cardiovascular, renal, and cognitive events [25].

Intracerebral hemorrhage: Hypertension can cause or contribute to intracerebral hemorrhage. The goal of BP management is to limit hemorrhage expansion. A randomized trial demonstrated that a strict BP control target of less than 140 mmHg within 6 h of onset did not improve survival but was associated with better functional outcomes [26]. A cluster randomized trial showed that strict BP control, when combined with other management strategies, reduced the risk of neurological sequelae [27]. Based on these findings, recent guidelines suggest that, in patients with acute spontaneous minor-to-moderate intracerebral hemorrhage, defined by a hematoma volume below 30 mL, and an initial systolic blood pressure of 150–220 mmHg, carefully titrated antihypertensive therapy should be initiated as early as possible, ideally within 2 h and no later than 6 h after symptom onset, to achieve an SBP below 140 mmHg. This target may subsequently be maintained for up to 7 days to limit hematoma expansion and blood-pressure variability, although a definitive improvement in functional outcomes has not been established [28].

Evidence supporting intensive BP lowering remains limited in patients presenting with an SBP above 220 mmHg with a large hematoma exceeding 30 mL, and those scheduled for hematoma evacuation, as these populations were underrepresented or excluded from major randomized trials. In these settings, cautious and closely monitored BP reduction is warranted because the optimal target and rate of reduction remain uncertain, and excessive lowering may increase the risks of neurological deterioration and systemic adverse events [28].

Intravenous calcium channel blockers, with the addition of urapidil, if necessary, are a suitable treatment option. Excessive and rapid reductions in systolic BP, exceeding 70 mmHg, should be avoided due to the risk of acute kidney injury and neurological deterioration [1].

I**schemic stroke:** During an acute ischemic stroke, up to 75% of patients present with BP above 140/90 mmHg [29]. This elevation may partly represent a compensatory hemodynamic response that helps maintain cerebral perfusion pressure and collateral blood flow to the ischaemic penumbra, particularly when cerebral autoregulation is impaired [30]. This acute hypertensive response is multifactorial, involving sympathetic activation, stimulation of the hypothalamic pituitary adrenal and renin angiotensin aldosterone systems, impaired baroreflex sensitivity, pain, anxiety, urinary retention and, occasionally, increased intracranial pressure [31].

Historically, the management strategy involved tolerating higher BP levels (up to 220/120 mmHg systolic/diastolic), as rapid BP reduction was thought to worsen neurological outcomes [32]. However, the advent of intravenous thrombolysis and mechanical thrombectomy has shifted this paradigm. In patients eligible for these therapies, the goal is to lower BP to <185/110 mmHg before thrombolysis and maintain it below 180/105 mmHg for the subsequent 24 h. Similarly, in patients undergoing mechanical thrombectomy (with or without intravenous thrombolysis), BP should be reduced to <180/105 mmHg before the procedure and maintained for the following 24 h. Therefore, patients with acute ischemic stroke and BP <180/105 mmHg within the first 72 h post-stroke do not appear to benefit from the introduction or reintroduction of antihypertensive therapy [28,33]. Furthermore, the 2025 European Stroke Organization guideline recommends against actively lowering systolic blood pressure below 140 mmHg during the first 24 h after successful mechanical thrombectomy, based on high-certainty evidence that this strategy may worsen neurological and functional outcomes [28]. Importantly, this recommendation does not imply that spontaneously occurring systolic blood pressure values below 140 mmHg should routinely be corrected.

Nicardipine or labetalol are commonly used for BP management in these patients, with close monitoring of BP every 15 min for the first two hours post-thrombolysis, every 30 min for the next 6 h, and hourly for the remaining 16 h of the first day [34]. In patients without reperfusion therapy, there is no clear benefit to actively reducing BP unless it exceeds 220/120 mmHg. In such cases, a slight reduction of approximately 10–15% over several hours may be deemed appropriate [1].

iii Renal Injuries

Numerous kidney diseases are associated with hypertension, and hypertension itself can impair renal function. Underlying kidney diseases may include chronic kidney disease, glomerulonephritis (such as vasculitis or cryoglobulinemia), macrovascular renal disease (bilateral renal artery stenosis or renal transplant artery stenosis), and microvascular disease (such as scleroderma renal crisis or thrombotic microangiopathy (TMA)). The presence of extrarenal signs, active urinary sediment (proteinuria, hematuria), and a targeted diagnostic workup can help identify the underlying cause of kidney disease.

In TMA, characterized by microangiopathic hemolytic anemia (with negative Coombs test and schistocytes), thrombocytopenia, and organ dysfunction, hypertension plays a central role, perpetuating endothelial injury and worsening prognosis [35,36]. Hypertension itself, particularly when it develops rapidly, can induce endothelial damage and lead to a TMA-like syndrome. While hypertension accounts for less than 5% of TMA cases [37], differentiating between TMA and malignant hypertension can be challenging, and the response to BP control can be a helpful diagnostic clue. In cases suggestive of TMA, specific laboratory investigations are crucial. These include ADAMTS13 activity, alternative complement pathway assessment, and evaluation for secondary causes of TMA.

Beyond treating the underlying cause, there are no specific recommendations for antihypertensive therapy in patients with hypertension and kidney disease, either in terms of drug choice or target BP. A general guideline is to reduce BP by approximately 25% over the first 24 h [38].

## Hypertensive disorders in pregnancy

Gestational hypertension or pregnancy-induced hypertension affects around 10% of pregnant women and usually manifests itself from the second trimester onwards. These are the symptoms of a placentation disorder, generally occurring between 12 and 16 weeks of amenorrhea. They may also be part of a pre-existing hypertension. Comprehensive management requires collaboration between gynecologists/obstetricians and nephrologists/cardiologists, which will not be detailed here.

Gestational hypertension is defined as a BP of ≥140/90 mmHg after 20 weeks of gestation in a previously normotensive woman which usually resolves within 6 weeks post-partum. Severe hypertension in pregnancy is defined as a BP of ≥160/110 mmHg for more than 15 min [1].

Preeclampsia is characterized by hypertension and proteinuria greater than 300 mg/day and is associated with complications such as eclampsia, HELLP syndrome (hemolytic anemia, elevated liver enzymes, and low platelet count), placental abruption, and adverse fetal outcomes. Diagnostic criteria and severity classification for preeclampsia are presented in Table 3 [39]. The pathophysiology of preeclampsia involves placental ischemia, leading to oxidative stress and maternal endothelial dysfunction. This explains the resolution of hypertension after delivery. However, women with preeclampsia have a higher long term cardiovascular risk compared to those with normotensive pregnancy [40], consequently, long-term clinical follow-up is recommended for this population [41].

**Table 3:** Classification of preeclampsia severity, according to [39].

Preeclampsia	▪ Blood pressure >140 and/or 90 mmHg after 20 weeks of pregnancy ▪ Proteinuria >0.3 g/24 h
Severe preeclampsia	▪ Systolic BP ≥ 160 and/or diastolic BP ≥110 mmHg, or uncontrolled hypertension ▪ Proteinuria >3 g/24 h ▪ Creatinine ≥90 µmol/L ▪ Oliguria (urine output ≤500 ml/24 h or ≤25 ml/h) ▪ Thrombocytopenia <100,000/mm^3^ ▪ Cytolysis (AST/ALT >2× upper limit of normal) ▪ Persistent or intense “tight” epigastric abdominal pain and/or right hypochondrial pain ▪ Chest pain, dyspnea, acute pulmonary edema ▪ Neurological signs: severe headache not responding to analgesics, persistent visual and/or hearing disturbances, brisk diffuse and hyperactive deep tendon reflex
Clinical or biological signs of seriousness in severe preeclampsia	▪ Systolic BP ≥180 and/or diastolic BP ≥120 mmHg ▪ Persistent or intense “tight” epigastric abdominal pain and/or right hypochondrial pain ▪ Respiratory distress, acute pulmonary edema ▪ severe headache not responding to analgesics, persistent visual and/or hearing disturbances, brisk diffuse and hyperactive deep tendon reflex ▪ HELLP syndrome ▪ Acute kidney injury

The primary goal of treatment is to control BP to reduce the risk of complications. In preeclampsia with hypertension, immediate reduction of systolic BP to <160 mmHg and diastolic BP to <105 mmHg is recommended using intravenous labetalol or nicardipine, with the aim of reaching this target within 150−180 min [1].

In cases of severe preeclampsia or signs of severity (Table 3), administration of intravenous antihypertensive drugs should be proposed (in addition to other therapies), with labetalol as first-line treatment in the absence of contraindications, with the aim of achieving extremely rapid control of BP < 180/120 mmHg within 10 min. If repeated boluses of labetalol (up to a cumulative dose of 220 mg) are ineffective, additional antihypertensive agents such as nicardipine or urapidil may be required [39].

Importantly, excessive BP lowering should be avoided during pregnancy to limit the risk of uteroplacental hypoperfusion. Although the optimal target remains uncertain, current guidelines generally recommend maintaining BP at approximately 130–135/80−85 mmHg [42].

## Malignant hypertension

i Definition

Historically, malignant hypertension was defined by a significant elevation in systolic BP, often exceeding 180 mmHg systolic and/or 120 mmHg diastolic, associated with grade III or IV hypertensive retinopathy based on the Keith-Wagener-Barker classification [43]. However, it has been recognized that malignant hypertension can even occur in the absence of retinal injuries [44,45].

This led to a broader definition emphasizing multi-organ involvement. Malignant hypertension is now defined as severe hypertension with evidence of damage to several (at least three) target organ, such as the kidneys, heart, brain, or retina, or the presence of microangiopathic hemolytic anemia, thrombocytopenia, and other systemic manifestations consistent with a TMA [46].

ii Epidemiology

Due to the widespread access to antihypertensive medications, the incidence and mortality rates linked to malignant hypertension have markedly decreased in comparison to the mid-20th century. However, the overall number of cases remains relatively stable. In European populations, the incidence of malignant hypertension is estimated to be 2–3 cases per 100,000 individuals. However, this incidence is significantly higher in individuals of African and Asian descent, exceeding 7 cases per 100,000. Given ongoing migration, the global incidence of malignant hypertension is expected to increase in developed countries [47].

The primary risk factors for malignant hypertension include uncontrolled essential hypertension, chronic kidney disease, and secondary causes of hypertension such as renovascular disease, endocrine disorders (pheochromocytoma, primary aldosteronism, Cushing's syndrome), and systemic vasculitis [48].

iii Pathophysiology

The hallmark of malignant hypertension is a rapid and severe elevation in BP [49]. When the body's autoregulatory mechanisms are overwhelmed, increased shear forces are transmitted to the microvasculature, leading to endothelial cell injury. The resulting endothelial dysfunction causes increased vascular permeability, allowing the leakage of plasma proteins, platelets, and fibrinogen into the vessel wall, which may eventually obstruct the capillary lumen and lead to tissue ischemia. Concomitantly, there is medial hypertrophy of arterioles and fibrinoid necrosis caused by the leakage of fibrin and other serum proteins through the necrotic vessel wall. This fibrinoid necrosis of small arteries (retinal, renal, cerebral), which may coexist with a true TMA, is a key feature of malignant hypertension [44]. The exposure of subendothelial proteins to the bloodstream triggers platelet adhesion and activation, as well as coagulation cascade activation, potentially contributing to a TMA.

The renin-angiotensin-aldosterone system (RAAS) plays a pivotal role in the pathogenesis and perpetuation of malignant hypertension [50,51]. Activation of the RAAS is triggered by several factors.

First, the marked elevation in BP is accompanied by an increase in natriuresis, called pressure natriuresis, that is independent of intravascular volume, through a direct renal mechanism [51]. Such enhanced sodium excretion leads to a reduction in effective volemia, thereby contributing to activation of the RAAS. In addition, the arterial vasoconstriction that occurs as an early compensatory response in malignant hypertension reduces the blood flow within the afferent renal arterioles. This decrease in renal perfusion serves as a key stimulus for increased renin production and secretion, a critical step in RAAS activation. The resulting increase in angiotensin II and aldosterone further elevates BP, perpetuating a vicious cycle.

Angiotensin II, through its effects on aldosterone secretion, promotes sodium retention and vasoconstriction [52]. Additionally, angiotensin II stimulates the production of pro-inflammatory cytokines and activates the transcription factor NF-κB, directly impairing endothelial function. Both angiotensin II and aldosterone contribute to vascular remodeling, characterized by intimal thickening and medial hypertrophy, further narrowing the vascular lumen and promoting fibrinoid necrosis.

iv Therapeutic management

The management of malignant hypertension aims to gradually bring BP to normal levels. While there is no consensus on the optimal rate of BP reduction, a general recommendation is to reduce mean arterial pressure by 20–25 % within 24 h, with a goal of normalization within 72 h. Rapid reductions in BP have been associated with an increased risk of stroke and mortality [53]. The primary intravenous agents used in the management of malignant hypertension include nicardipine, urapidil, and labetalol.

The evaluation of intravascular volemia in these situations is crucial. As described previously, natriuresis pressure may lead to intravascular volume depletion, and vasodilatory initiation may precipitate an excessive fall in BP and organs perfusion. This intravascular volume depletion can coexist with total body sodium excess and interstitial congestion [54,55]. In this context, intravascular hypovolemia refers to a reduction in effective circulating volume rather than necessarily to a deficit in total body water. However, hypovolemia is not always present. Volume status should therefore be carefully evaluated through an integrated assessment incorporating clinical examination [56], fluid balance and, when appropriate, bedside ultrasonography and dynamic indices [57,58]. Intravenous isotonic fluids should be considered only when clinically significant intravascular depletion is strongly suspected or documented and should be administered cautiously with frequent reassessment. Conversely, fluids should not be routinely administered and should generally be avoided in patients with overt systemic or pulmonary congestion, particularly those with hypertensive APE.

Initiation of renin-angiotensin-aldosterone system (RAAS) blockade with either an angiotensin-converting enzyme inhibitor or an angiotensin receptor blocker is essential. Low-dose therapy is recommended, with a preference for agents with shorter half-lives. Although some studies advocate for the early initiation of RAAS blockade, further research is necessary to determine the optimal timing [59]. The variable activation of the RAAS and the unpredictable therapeutic responses complicate the establishment of a standardized treatment protocol [50]. An initial rise in serum creatinine may be observed after commencing RAAS blockade; however, this should not require adjusting BP targets.

## Controversies in the definition and management of acute hypertension

The diagnostic classification of hypertensive emergencies as a framework for guiding management in the acute care setting remains a matter of ongoing debate [60,61]. This controversy stems from the fact that elevated BP in these situations is often a secondary response to an underlying acute illness, rather than a primary pathological process, as previously outlined. There is no evidence that rapid BP reduction in cases of asymptomatic acute hypertension in the emergency department improves clinical outcomes or prevents major cardiovascular events [62].

It is also essential to distinguish between the well-documented risks of chronic, uncontrolled hypertension [63] and the uncertain clinical significance of transient BP elevations in acute care settings. Importantly, randomized controlled trials evaluating cardiovascular outcomes in patients with hypertensive crises are lacking. Furthermore, recent observational studies suggest that treating asymptomatic BP elevation—especially with intravenous antihypertensive agents—may expose patients to unnecessary iatrogenic risks without proven benefit [[64], [65], [66], [67]].

## Conclusion

In acute settings, elevated BP must prompt a search for its cause. The varied clinical entities that present as hypertensive emergencies do not share a common pathophysiology justifying a uniform BP-lowering protocol. These underlying conditions are typically clinically evident and require management according to their specific guidelines for optimal patient care, which include comprehensive diagnostic and therapeutic strategies. Focusing solely on lowering BP, without treating the primary disorder, carries the risk of adverse outcomes.

After initial stabilization in an acute care setting, patients with hypertensive emergencies should be referred to a specialist. Specialized hypertension management is essential to complete the etiological assessment, adjust antihypertensive medications, and implement rigorous long-term follow-up to prevent further complications.

## Authors' contributions

All the authors concurred to draft the manuscript, all the authors revised the final version.

## Consent for publication

Not applicable.

## Ethics approval and consent to participate

Not applicable.

## Declaration of Generative AI and AI-assisted technologies in the writing process

The graphical abstract of our review was designed using the AU-tool Figure Labs (https://www.figurelabs.ai/).

## Funding

No funding.

## Availability of data and material

Not applicable.

## Declaration of competing interest

The authors declare to have no competing interests related to this work.
